# Global variations in funding and use of hemodialysis accesses: an international report using the ISN Global Kidney Health Atlas

**DOI:** 10.1186/s12882-024-03593-z

**Published:** 2024-05-08

**Authors:** Anukul Ghimire, Samveg Shah, Utkarsh Chauhan, Kwaifa Salihu Ibrahim, Kailash Jindal, Rumeyza Kazancioglu, Valerie A. Luyckx, Jennifer M. MacRae, Timothy O. Olanrewaju, Robert R. Quinn, Pietro Ravani, Nikhil Shah, Stephanie Thompson, Somkanya Tungsanga, Tushar Vachharanjani, Silvia Arruebo, Fergus J. Caskey, Sandrine Damster, Jo-Ann Donner, Vivekanand Jha, Adeera Levin, Charu Malik, Masaomi Nangaku, Syed Saad, Marcello Tonelli, Feng Ye, Ikechi G. Okpechi, Aminu K. Bello, David W. Johnson

**Affiliations:** 1 Division of Nephrology, Department of Medicine, University of Calgary, Calgary, AB Canada; 2 Department of Medicine, University of Alberta, Edmonton, AB Canada; 3 Division of Nephrology and Immunology, Faculty of Medicine and Dentistry, University of Alberta, Edmonton, AB Canada; 4 Nephrology Unit, Department of Medicine, Wuse District Hospital, Abuja, Nigeria; 5 Department of Internal Medicine, College of Health Sciences, Federal Capital Territory, Nile University, Abuja, Nigeria; 6 Bezmialem Vakif University School of Medicine, Istanbul, Turkey; 7 Department of Public and Global Health, Epidemiology, Biostatistics and Prevention Institute, University of Zurich, Zurich, Switzerland; 8 Renal Division, Brigham and Women’s Hospital, Harvard Medical School, Boston, MA USA; 9 Department of Paediatrics and Child Health, University of Cape Town, Cape Town, South Africa; 10 Division of Nephrology, University of Calgary, Calgary, AB Canada; 11 Division of Nephrology, Department of Medicine, College of Health Sciences, University of Ilorin, Ilorin, Nigeria; 12 Julius Center for Health Sciences and Primary Care, Julius Global Health, University Medical Center Utrecht, Utrecht University, Utrecht, The Netherlands; 13 Departments of Medicine & Community Health Sciences, Cumming School of Medicine, University of Calgary, Calgary, AB Canada; 14 Division of General Internal Medicine-Nephrology, Department of Medicine, Faculty of Medicine, Chulalongkorn University, Bangkok, Thailand; 15 Department of Medicine, John D. Dingell Veterans Affairs Medical Center, Wayne State University School of Medicine, Detroit, MI USA; 16 The International Society of Nephrology, Brussels, Belgium; 17 Population Health Sciences, Bristol Medical School, University of Bristol, Bristol, UK; 18 George Institute for Global Health, University of New South Wales (UNSW), New Delhi, India; 19 School of Public Health, Imperial College, London, UK; 20 Manipal Academy of Higher Education, Manipal, India; 21 Division of Nephrology, Department of Medicine, University of British Columbia, Vancouver, BC Canada; 22 Division of Nephrology and Endocrinology, The University of Tokyo Graduate School of Medicine, Tokyo, Japan; 23 Department of Medicine, University of Calgary, Calgary, AB Canada; 24 Canada and Pan-American Health Organization/World Health Organization’s Collaborating Centre in Prevention and Control of Chronic Kidney Disease, University of Calgary, Calgary, AB Canada; 25 Division of Nephrology and Hypertension, University of Cape Town, University of Cape Town, Cape Town, South Africa; 26 Department of Kidney and Transplant Services, Princess Alexandra Hospital, Brisbane, Queensland Australia; 27 Centre for Kidney Disease Research, University of Queensland, Princess Alexandra Hospital, Brisbane, Queensland Australia; 28 Australasian Kidney Trials Network, The University of Queensland, Woolloongabba, Queensland Australia; 29 Kidney and Hypertension Research Unit, University of Cape Town, Cape Town, South Africa; 30 Translational Research Institue, University of Queensland, Queensland Brisbane, Australia

**Keywords:** Arteriovenous fistulas, Central venous catheters, Dialysis, Global kidney Health Atlas, International Society of Nephrology, Kidney failure

## Abstract

**Background:**

There is a lack of contemporary data describing global variations in vascular access for hemodialysis (HD). We used the third iteration of the International Society of Nephrology Global Kidney Health Atlas (ISN-GKHA) to highlight differences in funding and availability of hemodialysis accesses used for initiating HD across world regions.

**Methods:**

Survey questions were directed at understanding the funding modules for obtaining vascular access and types of accesses used to initiate dialysis. An electronic survey was sent to national and regional key stakeholders affiliated with the ISN between June and September 2022. Countries that participated in the survey were categorized based on World Bank Income Classification (low-, lower-middle, upper-middle, and high-income) and by their regional affiliation with the ISN.

**Results:**

Data on types of vascular access were available from 160 countries. Respondents from 35 countries (22% of surveyed countries) reported that > 50% of patients started HD with an arteriovenous fistula or graft (AVF or AVG). These rates were higher in Western Europe (*n* = 14; 64%), North & East Asia (*n* = 4; 67%), and among high-income countries (*n* = 24; 38%). The rates of > 50% of patients starting HD with a tunneled dialysis catheter were highest in North America & Caribbean region (*n* = 7; 58%) and lowest in South Asia and Newly Independent States and Russia (*n* = 0 in both regions). Respondents from 50% (*n* = 9) of low-income countries reported that > 75% of patients started HD using a temporary catheter, with the highest rates in Africa (*n* = 30; 75%) and Latin America (*n* = 14; 67%). Funding for the creation of vascular access was often through public funding and free at the point of delivery in high-income countries (*n* = 42; 67% for AVF/AVG, *n* = 44; 70% for central venous catheters). In low-income countries, private and out of pocket funding was reported as being more common (*n* = 8; 40% for AVF/AVG, *n* = 5; 25% for central venous catheters).

**Conclusions:**

High income countries exhibit variation in the use of AVF/AVG and tunneled catheters. In low-income countries, there is a higher use of temporary dialysis catheters and private funding models for access creation.

**Supplementary Information:**

The online version contains supplementary material available at 10.1186/s12882-024-03593-z.

## Background

Hemodialysis (HD) can be initiated using various types of vascular access (arteriovenous fistula, arteriovenous graft, or central venous catheter). Data assessing outcomes between patients using different types of vascular access are often confounded by selection bias [1]. However, compared to central venous catheters (CVC), arteriovenous fistulas (AVF) are reported to be associated with enhanced dialysis quality and reduced risk of infections [2, 3].

Selecting the ideal type of vascular access for any given patient is complex and depends on numerous factors, such as patient preferences, vascular health and anatomy, ability of a patient to tolerate needling pain and high-outflow states related to fistulas, urgency of dialysis, and availability of specialist surgeons [4]. Further, despite the perceived superiority of AVF over CVC, patients utilizing AVF may have less satisfaction relating to quality-of-life indices compared to those using CVC [5, 6]. Newer guidelines put more emphasis on patients’ needs and preferences when choosing an access type, described as a patient’s “End Stage Kidney Disease Life-Plan” [3].

The uptake of “End Stage Kidney Disease Life-Plan” among countries with less resources may be limited. Funding models for HD vary between countries and range from private models (that are dependent on patients paying out of pocket) to fully public systems (that are free at the point of delivery). Given the projection that 5.4 million people worldwide will need kidney replacement therapy (KRT) by 2030 [7, 8], there is value in understanding the global differences in HD vascular access use and funding models. This will be of the utmost importance in low- and lower-middle income countries where the availability of services to initiate HD are limited already [8, 9] and up-to-date and comprehensive data is needed to guide policy change to increase the availability and affordability of kidney care.

Further, discrepancies in financial funding models for the creation of vascular access using contemporary data are not well described. We used data from the third iteration of the International Society of Nephrology Global Kidney Health Atlas (ISN-GKHA) [10] to highlight the current status and differences across countries and world regions regarding (1) utilization of a functioning arteriovenous access (AVF and arteriovenous grafts) vs. tunneled CVC to initiate HD (2), prevalence of temporary catheters used for HD initiation, and (3) funding models utilized for vascular access creation.

## Methods

Information regarding the development and validation of the ISN-GKHA have been described previously [10, 11]. Important aspects of the ISN-GKHA methods relevant to this report are summarized below.

### Survey design

The ISN-GKHA was conducted across countries in all ISN regions. A non-probability, purposive sampling approach was used to identify potential respondents in each country. National and regional nephrology leaders, policymakers, and patient advocates in each country were identified through ISN contacts and selected to participate in the survey. The survey was completed via an online portal (REDCap; www.project-redcap.org) between June and September 2022.

Countries from these regions were stratified into income groups (low-, low-middle, upper-middle, and high income) for analysis using the 2022 World Bank income stratification for countries. A complete review of the countries chosen, their ISN regional classification, and their World Bank Income classifications has been published previously [10]. The following 10 ISN regions were used for analysis: Africa, Eastern and Central Europe, Latin America, Middle East, North America and the Caribbean, North and East Asia, Oceania and South East Asia (OSEA), Newly Independent States (NIS) and Russia, South Asia, and Western Europe.

Survey questions were designed with input from relevant experts, members of the ISN leadership, and regional and national nephrology leadership. The overall survey was designed to provide relevant information on the availability, accessibility, quality, and affordability of kidney care programs using a framework based on the World Health Organization’s health system building blocks. The building blocks focused on health financing, service delivery, access to essential medicines and health technologies, health information systems, workforce, and health leadership [9]. For this work, we focus on the sections of the survey relating to the availability of different types of HD accesses (AVF/AVG, CVC, and tunneled catheters) utilized for initiating HD across countries. How these access types were funded (public funds and free, public funds with some payments, private health insurance, private and out-of-pocket, or a mix of public and private funds) were also assessed in the survey for each HD access type.

The survey was made available in English, French, and Spanish with non-English responses translated into English.

### Definitions

AV (arteriovenous) access was defined as a structure that connects a vein to an artery for the purpose of providing dialysis. These were subdivided into an AV Fistula (AVF, a surgical connection of a vein to an artery) or an AV Graft (AVG, a surgical connection of a vein to an artery using a synthetic or biological tube). A permanent or tunneled CVC referred to a subcutaneously tunnelled catheter placed in the central veins and used for the purposes of facilitating dialysis whereas a temporary CVC was not tunnelled and placed directly into the central veins. Although the term “non-tunnelled CVC” may be preferred to “temporary CVC” by some, the ISN-GKHA questions did refer to these catheters as “temporary CVC” and so we will continue to use this term throughout this manuscript for the sake of consistency. The dominant form of vascular access in each country was defined as the most commonly used vascular access at the initiation of HD. For countries that had similar reported use of two or three forms of access, they were defined as having “co-dominant” or “relatively equal use” forms of vascular access, respectively. As an example, if a country reported use of AVF/AVG 51–75%, tunnelled CVC 11–50%, and temporary CVC 1–10% respectively, the dominant form of access was described as AVF/AVG. If a country instead reported use of AVF/AVG 11–50%, tunnelled CVC 11–50%, and temporary 1–10%, a co-dominant form between AVF/AVG and tunneled CVC was used. Countries were stratified into high-income (HICs), upper-middle income (UMICs), lower-middle income (LMICs), and low-income (LICs) based on the World Bank classification. Regional classification of countries was based on the 10 ISN region classifications outlined above [11]. Publicly funded and free models of funding were those in which patients had no net cost at the point of delivery with funding/reimbursement offered through a government agency. Privately funded payment systems referred to those that were out-of-pocket payments provided by patients at the point of delivery. Health insurance providers referred to non-governmental agencies that provided funding at the point of delivery. An option for non-governmental agencies, communities, or other funding models was also presented.

### Data handling and analysis

Data were recorded and cleaned in Microsoft Excel and then collected into a single file. Given that the unit of analysis was country and there were multiple respondents from one country, the national project leader was responsible for reviewing data, following up on discrepancies, and ultimately providing a final response for the country. Furthermore, a project leader was identified at the regional level to organize and follow up on the national responses, vet and review data, and provide a final answer for the data in their region for analysis. Data were reported as counts and percentages of each ISN region and World Bank income group. The data were analyzed using STATA 17 software (Stata Corporation, 2021). Variations in types of accesses used for dialysis initiation and funding models across regions and country income levels were presented using heatmaps and bar plots.

## Results

### Survey respondents

Of the 167 countries that participated in the survey, data on vascular access types for HD initiation were available from 166 countries (99.4%), classified as LICs (12.0%; *n* = 20), LMICs (27.1%; *n* = 45), UMICs (22.9%; *n* = 38), and HICs (38.0%; *n* = 63) (Tables 1 and 2). Data on prevalence of different vascular access modalities and proportion of patients who received dialysis education are also presented in the Additional files (Additional Figs. 1 and 2).

**Table 1:** Funding modules for the creation of central venous catheters [*N* (%)]

	Publicly funded by government and free at the point of delivery		Publicly funded by government but with some fees at the point of delivery		A mix of publicly funded (whether or not publicly funded component is free at point of delivery) and private systems		Solely private and out of pocket		Solely private through health insurance providers		Multiple systems - programs provided by government, NGOs, and communities		Other		Total
Overall	69	(42)	27	(16)	38	(23)	17	(10)	3	(2)	9	(5)	3	(2)	166
ISN region:															
Africa	7	(17)	9	(22)	11	(27)	10	(24)	1	(2)	2	(5)	1	(2)	41
Eastern & Central Europe	14	(88)	1	(6)	1	(6)	0	(0)	0	(0)	0	(0)	0	(0)	16
Latin America	6	(27)	1	(5)	13	(59)	0	(0)	0	(0)	2	(9)	0	(0)	22
Middle East	6	(55)	3	(27)	0	(0)	0	(0)	1	(9)	1	(9)	0	(0)	11
NIS & Russia	3	(30)	4	(40)	0	(0)	2	(20)	0	(0)	1	(10)	0	(0)	10
North America & the Caribbean	4	(33)	4	(33)	3	(25)	1	(8)	0	(0)	0	(0)	0	(0)	12
North & East Asia	3	(50)	2	(33)	1	(17)	0	(0)	0	(0)	0	(0)	0	(0)	6
OSEA	4	(22)	2	(11)	7	(39)	3	(17)	0	(0)	1	(6)	1	(6)	18
South Asia	2	(25)	1	(13)	2	(25)	1	(13)	0	(0)	1	(13)	1	(13)	8
Western Europe	20	(91)	0	(0)	0	(0)	0	(0)	1	(5)	1	(5)	0	(0)	22
World Bank Groups:															
Low income	3	(15)	5	(25)	4	(20)	5	(25)	0	(0)	2	(10)	1	(5)	20
Lower-middle income	7	(16)	8	(18)	13	(29)	11	(24)	1	(2)	3	(7)	2	(4)	45
Upper-middle income	15	(39)	7	(18)	12	(32)	1	(3)	1	(3)	2	(5)	0	(0)	38
High income	44	(70)	7	(11)	9	(14)	0	(0)	1	(2)	2	(3)	0	(0)	63

Abbreviations: ISN, International Society of Nephrology; NGOs, non-governmental organizations; NIS, Newly Independent States; OSEA, Oceania and South East Asia

**Table 2:** Funding modules for the creation of arteriovenous fistulas and grafts [N (%)]

	Publicly funded by government and free at the point of delivery		Publicly funded by government but with some fees at the point of delivery		A mix of publicly funded (whether or not publicly funded component is free at point of delivery) and private systems		Solely private and out of pocket		Solely private through health insurance providers		Multiple systems - programs provided by government, NGOs, and communities		Other		Total
Overall	64	(39)	25	(15)	39	(23)	22	(13)	3	(2)	8	(5)	5	(3)	166
ISN region:															
Africa	7	(17)	5	(12)	10	(24)	13	(32)	1	(2)	3	(7)	2	(5)	41
Eastern & Central Europe	11	(69)	2	(13)	3	(19)	0	(0)	0	(0)	0	(0)	0	(0)	16
Latin America	6	(27)	1	(5)	12	(55)	1	(5)	0	(0)	2	(9)	0	(0)	22
Middle East	5	(45)	3	(27)	2	(18)	0	(0)	1	(9)	0	(0)	0	(0)	11
NIS & Russia	2	(20)	5	(50)	0	(0)	2	(20)	0	(0)	1	(10)	0	(0)	10
North America & the Caribbean	4	(33)	4	(33)	2	(17)	2	(17)	0	(0)	0	(0)	0	(0)	12
North & East Asia	2	(33)	3	(50)	1	(17)	0	(0)	0	(0)	0	(0)	0	(0)	6
OSEA	4	(22)	2	(11)	6	(33)	3	(17)	0	(0)	1	(6)	2	(11)	18
South Asia	3	(38)	0	(0)	3	(38)	1	(13)	0	(0)	0	(0)	1	(13)	8
Western Europe	20	(91)	0	(0)	0	(0)	0	(0)	1	(5)	1	(5)	0	(0)	22
World Bank Groups:															
Low income	2	(10)	2	(10)	3	(15)	8	(40)	0	(0)	3	(15)	2	(10)	20
Lower-middle income	6	(13)	8	(18)	14	(31)	12	(27)	1	(2)	1	(2)	3	(7)	45
Upper-middle income	14	(37)	6	(16)	13	(34)	2	(5)	1	(3)	2	(5)	0	(0)	38
High income	42	(67)	9	(14)	9	(14)	0	(0)	1	(2)	2	(3)	0	(0)	63

Abbreviations: ISN, International Society of Nephrology; NGOs, non-governmental organizations; NIS, Newly Independent States; OSEA, Oceania and South East Asia

### Dominant access for dialysis initiation

**Fig. 1:**
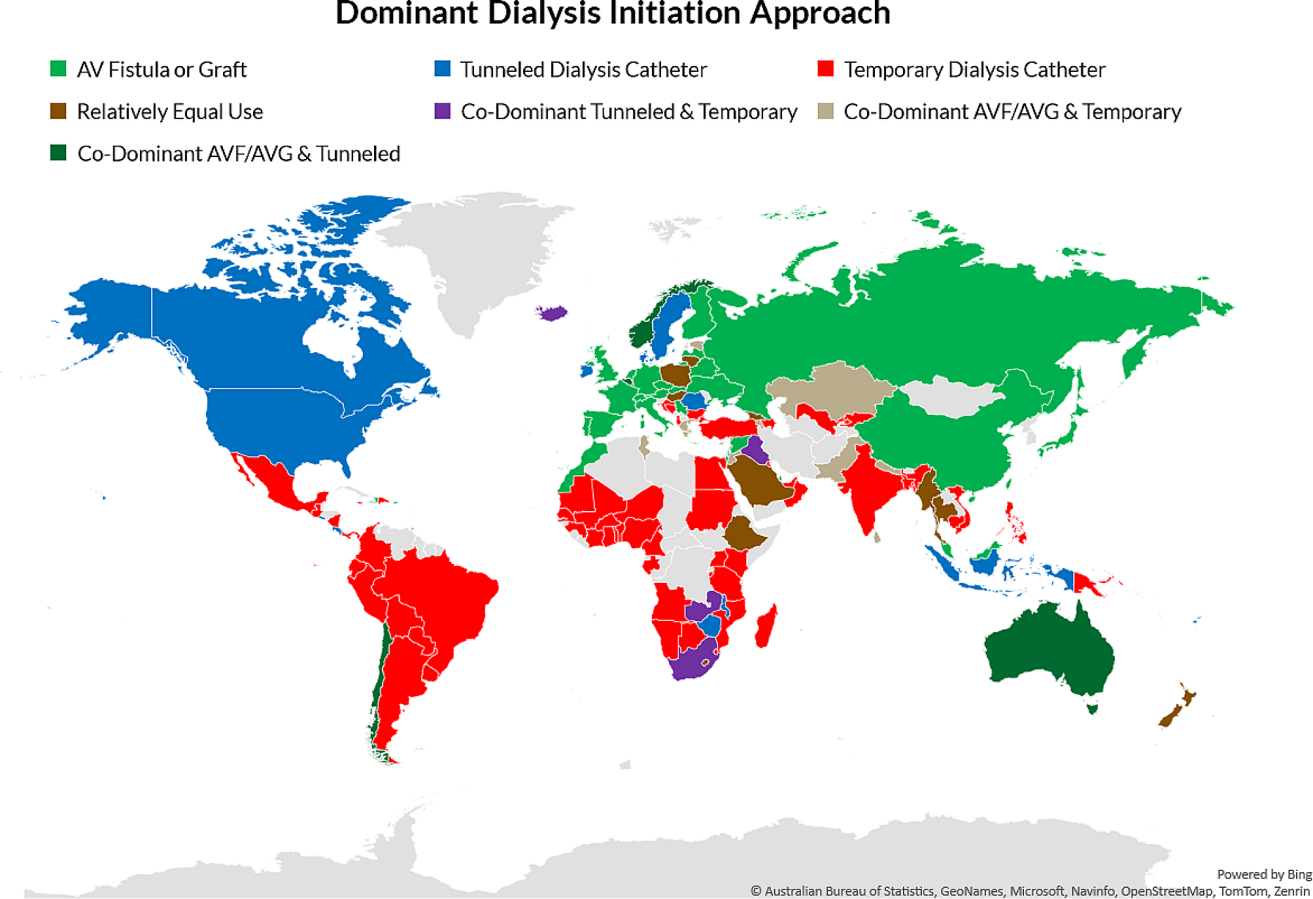
Geographic heatmap for the dominant approach to dialysis initiation by country. Data was generated from survey responses highlighting the utilization of various techniques. As responses were reported in ranges [0-10, 11-50, 50-74, 75%+], multiple countries were considered "co-dominant" if there were an equal reported range for two approaches to initiate dialysis. AV, arteriovenous; AVF, arteriovenous fistula; AVG, arteriovenous graft

When grouped based on highest reported usage of HD vascular accesses, 79% of countries (*n* = 123) had one HD access type that was used by > 50% of patients in the country while 7% of countries (*n* = 10) reported having relatively similar rates of AVF/AVG, tunneled catheters, and temporary catheters used for HD initiation (Fig. 1, Additional Table 4). Countries in North America tended to have tunneled dialysis catheters as the dominant access for HD initiation, whereas Western Europe, China, and Russia used AVF/AVG as the dominant access modality for HD initiation in most patients. In Africa, Latin America, and South Asia, temporary catheters were frequently used to initiate HD while the OSEA region reported similar rates of AVF/AVG and tunneled catheter use (Fig. 1).

### Use of functioning vascular access to initiate hemodialysis

**Fig. 2:**
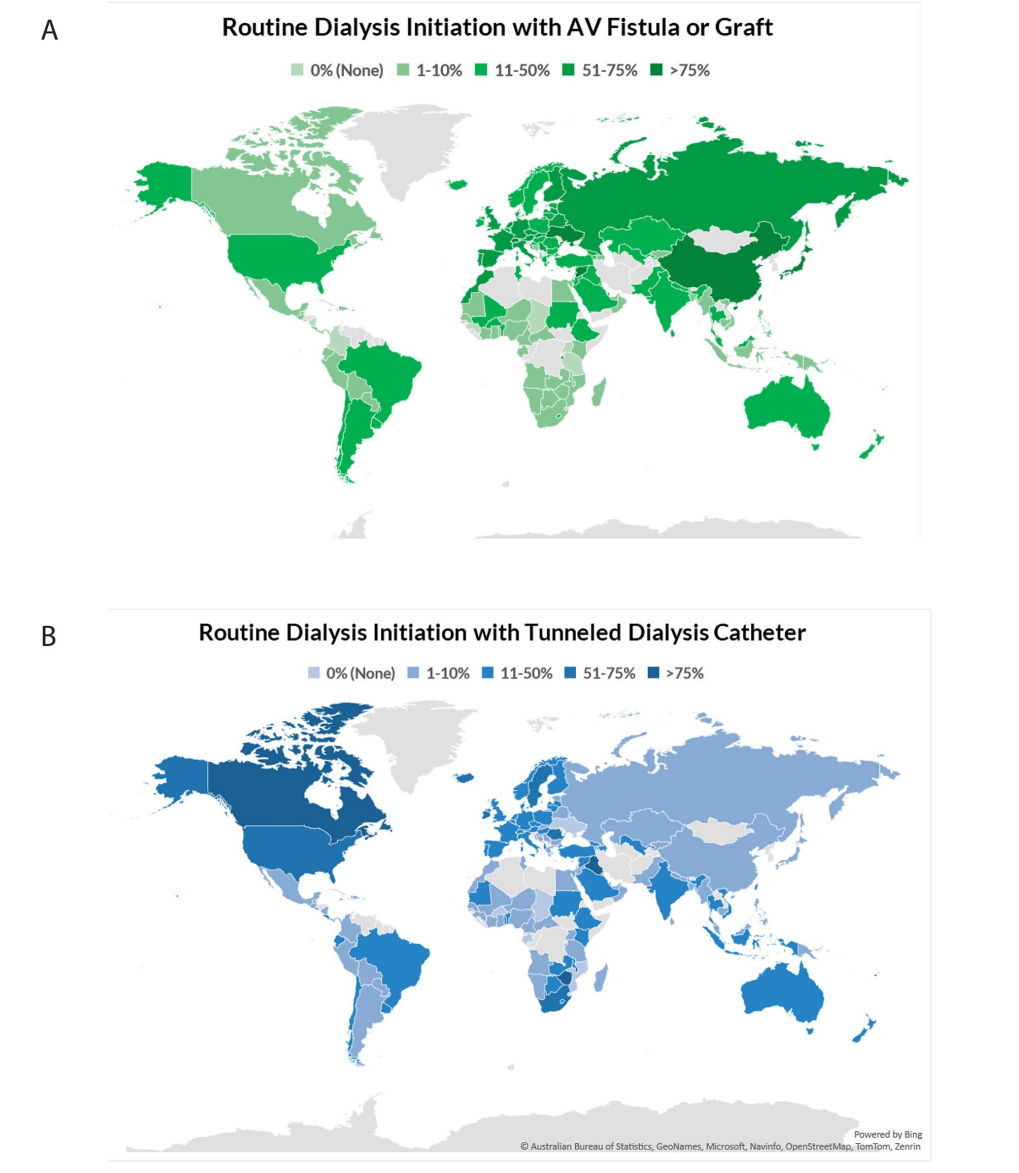
Geographic heatmaps demonstrating international variation in the method of dialysis initiation. (A) Proportion of patients routinely starting dialysis with AV fistula or graft. (B) Proportion of patients routinely starting with a tunneled dialysis catheter. Answers range from 0% (None), 1–10% (Few), 11–50% (Some), 51–75% (Most), and > 75% (Almost all). AV, arteriovenous

Overall, 22% of countries (*n* = 35) had > 50% of patients starting HD with a AVF or AVG (Table S1, Fig. 2A). Among the ISN regions, Western Europe (64%; *n* = 14) and North & East Asia (67%; *n* = 4) had the highest proportion of > 50% of patients starting HD with AVF/AVG while OSEA (6%; *n* = 1) had the lowest (Fig. 2A). Of the 35 countries that reported that > 50% of patients used AVF/AVG to initiate dialysis, 83% (*n* = 24) were from HICs (Additional Table 1). Of the 71 countries that reported no (0%) or few (1–10%) patients starting dialysis with AVF/AVG, 55% (*n* = 39) were from LICs or LMICs (Additional Table 1). In Canada, only few (1–10%) patients routinely started dialysis with AVF/AVG, and the United States of America (USA) reported that some (11–50%) of their patients started dialysis with AVF/AVG. However, in China, Japan, Republic of Korea, Switzerland, and Andorra, almost all (> 75%) of patients started dialysis with AVF/AVG and most (51–75%) patients on HD from the Russian Federation started dialysis with AVF/AVG.

### Use of tunneled dialysis catheter to initiate hemodialysis

We found that 15% of countries (*n* = 24) had > 50% of patients start HD with a tunnelled dialysis catheter (Additional Table 2 / Fig. 2B). North America & the Caribbean had the highest proportion of countries in which almost all patients started HD with a tunnelled dialysis catheter (50%; *n* = 6) (Fig. 2B). Only 1 country (17%) in North & East Asia and 6 (27%) countries in Western Europe reported that most patients started HD with a tunnelled dialysis catheter. There were no countries in Eastern and Central Europe, Latin America, NIS & Russia, North & East Asia, South Asia, or Western Europe that reported that almost all patients started dialysis with a tunneled dialysis catheter. Of the 24 countries that reported > 50% patients started HD with a tunneled dialysis catheter, 83% (*n* = 20) were in HIC or UMIC categories (Additional Table 2). Regarding some country specific data, Zimbabwe, Iraq, and Fiji reported that almost all patients started dialysis using a tunneled catheter whereas China, Japan, and the Russian Federation reported that only few patients started dialysis using a tunneled catheter.

### Use of temporary dialysis catheter to initiate hemodialysis

**Fig. 3:**
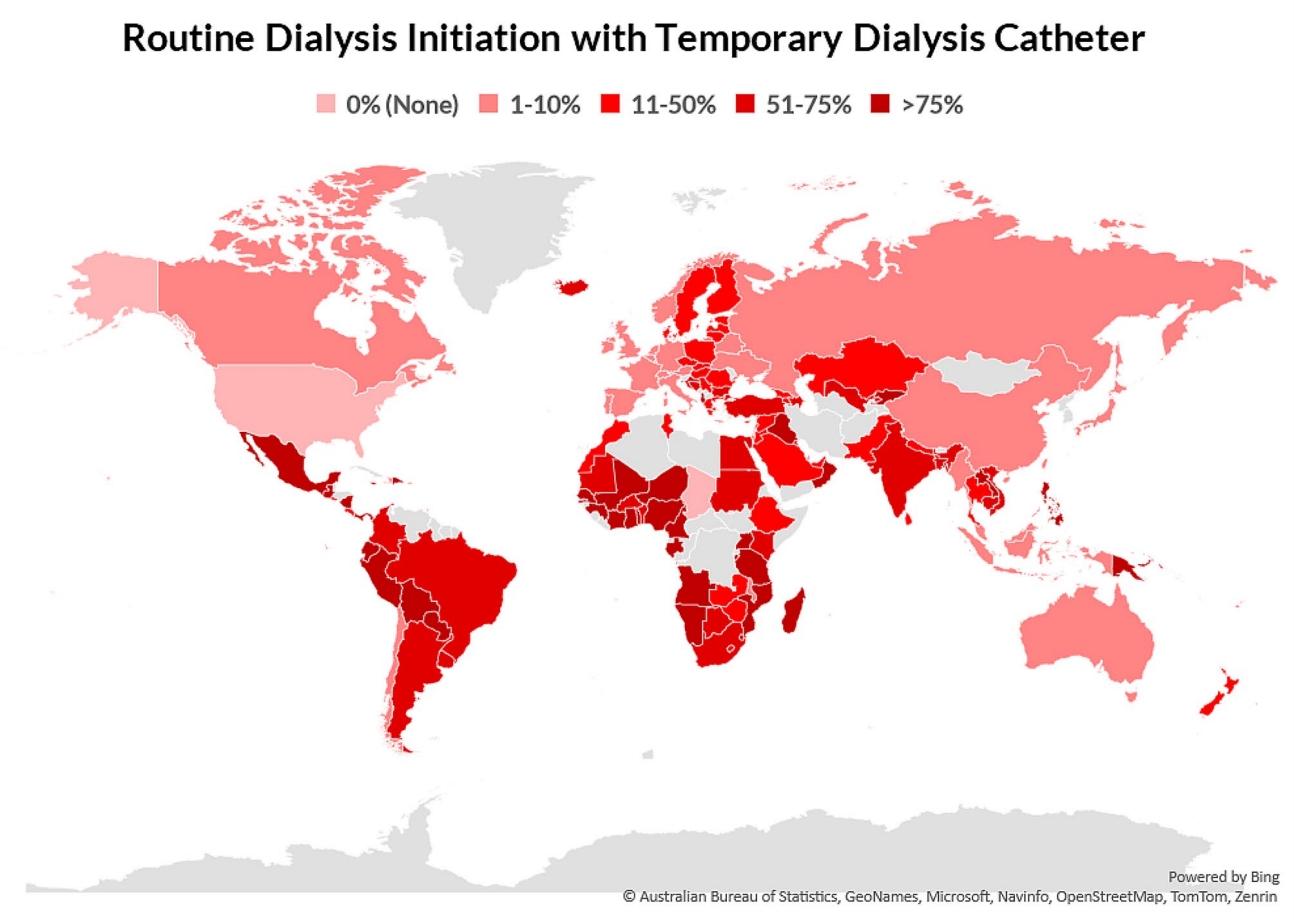
Geographic heatmap demonstrating international variation in the proportion of patients routinely starting dialysis with a temporary dialysis catheter. Answers range from 0% (None), 1–10% (Few), 11–50% (Some), 51–75% (Most), and > 75% (Almost all)

We found that 44% of countries (*n* = 71) had > 50% of patients starting HD with a temporary dialysis catheter (Fig. 3). Among these countries, 75% (*n* = 30) were from Africa and 67% (*n* = 14) from Latin America. Among countries from Western Europe and North & East Asia, 73% (*n* = 16) and 67% (*n* = 4) reported that only few patients started dialysis using a temporary dialysis catheter respectively. However, compared to LICs where 50% (*n* = 9) of countries reported almost all patients started dialysis with a temporary dialysis catheter, only 6% (*n* = 4) of HICs reported that almost all patients started HD with a temporary catheter (Additional Table 3). Almost all countries in South Asia, where data were available, reported that some or most patients initiated dialysis with a temporary catheter, except for Maldives where only few patients initiated dialysis with a temporary catheter.

### Funding for vascular access creation

**Fig. 4:**
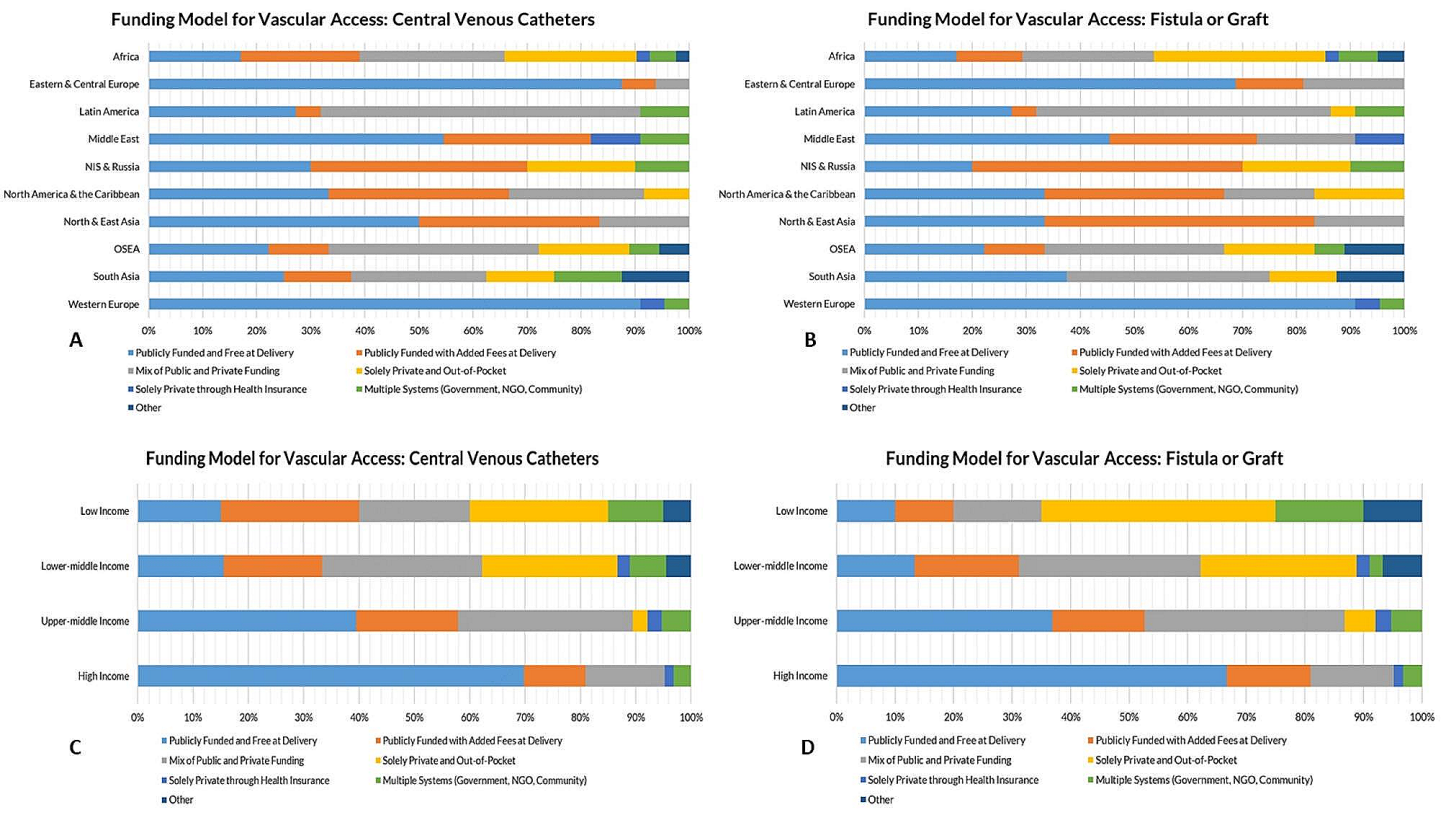
Healthcare systems coverage for surgical services for Kidney Replacement Therapy. Funding models for the creation of central venous catheters are shown as bar plot segments representing a fraction of countries in each International Society of Nephrology region **(A)** and in each category of World Bank Income Group **(C)**. Funding models for the creation of arteriovenous fistulas and grafts are shown as bar plot segments representing a fraction of countries in each International Society of Nephrology region **(B)** and in each category of World Bank Income Group **(D)**. NGO, non-governmental organization; NIS, Newly Independent States; OSEA, Oceania and South East Asia

For the insertion of CVC, funding was free at the point of delivery and reimbursed through public funding in 42% (*n* = 69) of countries. Funding for CVC insertion was free at the point of delivery and reimbursed through public funding in 70% (*n* = 44) of HICs and only 15% (*n* = 3) of LICs. Public funding that was free at point of delivery was used for CVC insertion in 91% (*n* = 20) of Western European countries, 88% (*n* = 14) of Eastern & Central Europe countries, but only 17 (*n* = 7) of countries in Africa. Funding for CVC was solely private and out-of-pocket in 25% (*n* = 5) and 24% (*n* = 11) of LICs and LMICs, respectively, compared to 3% (*n* = 1) of UMIC. No HICs used this funding model for CVC insertion (Table 1; Fig. 4A and B).

For the creation of fistulas or grafts, funding was free at the point of delivery and reimbursed through public funding in 39% (*n* = 64) of countries. This model was used for AVF/AVG creation in 67% (*n* = 42) of HICs and 10% (*n* = 2) of LICs. By ISN regions, public funding and free at point of delivery was used to cover the cost of AVF/AVG creation in 91% (*n* = 20) of countries in Western Europe, 69% (*n* = 11) of countries in Eastern & Central Europe, and only 17% (*n* = 7) of countries in Africa. Funding for AVF/AVG was solely private and out-of-pocket in 40% (*n* = 8) and 27% (*n* = 12) of LICs and LMICs, respectively, whereas this was the case in only 5% (*n* = 2) of UMICs and was absent in HIC as a funding model (Table 2; Fig. 4C and D).

## Discussion

Our study adds to the evolving literature describing global disparities in kidney care using data from the ISN-GKHA [10, 12–16]. To the best of our knowledge, this study provides the most up-to-date data regarding global variations in types of vascular accesses used for HD initiation across all countries and regions of the world. It is also one of the first attempts at describing differences in the use of functioning AVFs/AVGs, tunneled dialysis catheters, and temporary (or non-tunneled) dialysis catheters across all world regions. Among HICs, AVFs/AVGs and tunneled CVCs were used most often. AVF/AVG use was greater in Western Europe and North & East Asia but lower in North American and the Caribbean countries, such as Canada and USA, where tunneled dialysis catheters were frequently used for dialysis initiation. In Latin America and Africa, temporary dialysis catheters were the dominant form of access used at dialysis initiation. We found significant variations in the funding models used for vascular access creation, with HICs and UMICs using public funding models more often and LICs and LMICs relying more on private, out-of-pocket methods.

Our data showed substantial variation in the use of AVF/AVG among HICs. The rates of AVF and AVG creation were much higher in countries such as China, Japan, Russia, and many Western European countries, when compared to other HICs such as Canada and the United States. This suggests that differences in the uptake of AVF/AVG may be accounted for by other factors outside of availability of resources and funding models.

Varying local patient demographics may be one factor accounting for differences in vascular access utilization [17–21]. AVF require a period of maturation and may be more prone to failure in patients with peripheral arterial disease (PAD), coronary artery disease, dyslipidemia, diabetes and advanced age [22]. Previous work from the Dialysis Outcomes and Practice Patterns Study (DOPPS) [23] comparing dialysis cohorts between world regions in 2015 showed that the primary cause of kidney failure (KF) was glomerulonephritis in Russia (38.8%) and Japan (39.6%), whereas diabetes (43%) was the most common etiology of KF in the North American cohort. North American patients were typically older and more often had PAD than patients from other regions [23]. Similarly, a DOPPS study from China (2012–2015) showed that the most common cause of KF was chronic glomerulonephritis (45.9%), PAD prevalence was relatively low, and 88% of patients on HD used fistulas [24]. Thus, this would suggest that differences in the demographics of patients on dialysis—especially as it relates to risk of fistula failure—could partially explain the reported differences in AVF/AVG use in our survey.

Country specific healthcare related factors may also lead to differences in vascular access use rates. For example, we found that Canada had much lower reported rates of AVF to initiate dialysis when compared to other HICs such as the USA and United Kingdom, where diabetes, renovascular disease, and advanced age are shared common features of the dialysis cohorts [25–27]. A previous Canadian survey study showed that the lower rates of AVF use may have been due to a combination of long wait times to access surgeons and lower rates of referrals to interventionalists to assist with AVF maturation [28]. This same study also found that the most common cited reason for not using AVF was primary failure. Thus, it follows that the combination of high rates of AVF maturation failure, long wait times to access surgeons, and low referral rates to interventionalists may influence the overall low rates of AVF/AVG use in Canada [29]. Further, Canadian practitioners are not compensated for the use of a particular access to initiate dialysis, unlike in the USA where there are certain financial reimbursements for AVF utilization [30].

Our survey found that there is a wide variation in tunneled catheter use, with higher rates in HICs. It is worth noting that there has been a recent paradigm shift regarding the perceived superiority of fistulas over catheters. The Fistula First Initiative (FFI) was introduced in 2003 as a means of promoting AVF use, given the perceived superiority of AVF compared to CVC [31]. Data from the both the DOPPS [32] and United States Renal Data System [33, 34] have shown that AVF use in the United States has risen after the introduction of FFI [33, 34]. This concept later evolved to the “Fistula First Catheter Last” initiative to emphasize the importance of both increased AVF uptake and reduced catheter use [35].

More recently however, the benefits of prioritizing fistulas have been called into question. It has been shown that the perceived mortality benefits of AVF over CVCs are largely due to patient selection, rather than the type of access used. This suggests that there is selection bias present in studies looking at clinical outcomes between patients using AVF vs. CVC [36] and the perceived benefits of using AVF or AVG are not supported by robust, unbiased data. Further, there has been emphasis on maintaining quality of life and comfort for patients undergoing HD. This is especially true to elderly patients who seem to be more bothered by the fistula related pain, bruising, bleeding, and swelling and may prefer the use of CVCs [6]. Elderly patients are also more likely to have co-morbidities that increase their risk of fistula maturation failure and are more likely to die before the fistula can be used, suggesting that CVC may be preferred in cases where the perceived benefits of AVF don’t outweigh the risks [37]. Thus, recent international guidelines have promoted a more tailored and patient-centered focus for choosing vascular access for patients on HD [3].

It is worth noting that the recommendations for vascular access in China were published after the most recent National Kidney Foundation - Kidney Disease Outcome Quality Initiative guidelines (2019), and still recommend AVF as access of choice in the Chinese dialysis population [38]. Although they do acknowledge the shift from a “fistula-first” to “patient first” approach in the international guidelines, they still recommend AVF use for their population. However, it was stated that the recommendations were general, and clinicians needed to make treatment decisions based on the individual patient. On the contrary, Canadian providers may have less enthusiastic views about AVF being the first choice of access. In a survey-based study of Canadian nephrologists, surgeons, and vascular access nurses, 27% of providers were neutral or disagreed with the statement, “AVFs are the first choice of access for all patients.” [28]. Thus, differences in AVF use may be guided by attitudes of local practitioners such that heterogeneity in a “fistula first” vs. “patient-centered” approach between different countries is an important consideration.

Unfortunately, a patient-centered approach may not be feasible in low-income regions of the world where critical workforce shortages necessary for creating vascular accesses and providing these patient-centered options are limited [13]. Our study showed that a large portion of LICs, especially in Africa and Latin America, are using the less-preferred temporary dialysis catheters to initiate HD. Placing temporary HD catheters does not require access to surgeons or special radiology suites and so is less resource intensive. It does however come with higher risks of infections and complications and is less preferred overall if another form of vascular access is available [39, 40]. Thus, the high rates of temporary catheter use in these countries may be more indicative of lower access to interventionalists, operating suites, financial capacity for the creation of AVF/AVG or tunneled catheters, and also lower access to other forms of KRT, such as peritoneal dialysis and kidney transplantation [41, 42].

The higher rates of temporary catheter use may also reflect the higher rates of late presenting patients in LICs. Patients in lower income countries more often present late with advanced chronic kidney disease (CKD) and acute kidney injury thereby necessitating the immediate use of dialysis with no time for AVF/AVG creation or maturation [40, 43, 44]. The high rates of late presenting diseases in resource strained countries are further compounded by issues surrounding: (1) disproportionality high rates of incidence and prevalence of non-communicable diseases, such as CKD in lower income countries; (2) significant workforce shortages for the growing population of patients with CKD [13]; (3) a lack of access to laboratory tools (such as serum creatinine and urine protein quantification) that enables early CKD identification and treatment [45]; and (4) limited public funding and government sponsored financial aid for healthcare expenditures [41].

Our survey showed that patients in lower income- and lower middle-income countries relied more on private, out of pocket funding and had less public funding available for vascular access creation. This adds to the previous work that has shown the dominance of private, out of pocket funding models in LICs for many aspects of kidney care including dialysis, non-dialysis CKD, and kidney transplantation [46]. These same countries often report poor healthcare infrastructure, fragmented oversight with individual hospitals providing their own individual leadership (whereas in HICs there was more regional/state oversight) [46]. Ultimately, this manifests as patients not only having less access to vascular access options, but to less KRT in general, and increases the risk of premature mortality from untreated KF. For example, it was estimated that the shortfall for provision of KRT in the public sector of the Eastern Cape of South Africa was over 8600 patients [47]. Similarly, it is estimated that the majority of patients in India who develop KF do not end up seeing a nephrologist and, of those that do, 10% are not able to afford KRT [48]. This pattern of unavailable kidney care in LICs is alarming, and calls for further work to develop infrastructure, health policy, workforce development, and research in the developing world. Expressed otherwise, global efforts should shift away from deciding which vascular access is best, and towards providing access to KRT including availability of dialysis in developing nations. Thus, a “fistula-first” approach should be replaced with an “equitable access to dialysis” approach.

There are a few limitations to our survey. Our survey used opinion-based data which were subjective and prone to bias from the respondents. Another limitation arose from the use of median data to represent regions, which may have been skewed by other countries within the region and therefore not faithfully represented the true picture. This was addressed by using country specific data in the creation of the global maps to emphasize the granular data that were available. Lastly, we were not able to comment on differences between patients in countries where both public and private healthcare sectors exist. This may be important given noted health disparities between patients in these two cohorts as it relates to dialysis care [47].

In summary, our analysis highlights that, in HICs, there are regional differences in the use of AVF/AVG and tunneled dialysis catheters. This may be accounted for by differences in patient demographics, health system financing, and local healthcare related factors (including practice culture) that promote the use of one type of vascular access over the other. In LICs, temporary catheters and private, out of pocket funding are frequently being utilized and reflect the ongoing challenges of these countries with healthcare organization, financing, and workforce shortages. Further work is needed to find solutions in: (1) understanding the reasons for differences in vascular access modalities used to initiate HD, especially in countries within the same income category; (2) defining the ideal type of vascular access for each patient/cohort; and (3), allowing patients more options for vascular access and kidney care in resource limited countries. Global efforts to build health care capacity in the developing world are needed.

### Electronic supplementary material

Below is the link to the electronic supplementary material.

Supplementary Material 1

Supplementary Material 2

## Data Availability

The datasets used and analysed during the current survey are available from the corresponding author on reasonable request.

## Abbreviations

AVF: Arteriovenous fistula
AVG: Arteriovenous graft
CKD: Chronic kidney disease
CVC: Central venous catheter
DOPPS: Dialysis Outcomes and Practice Patterns Study
ESKD: End stage kidney disease
FFI: Fistula First Initiative
GKHA: Global Kidney Health Atlas
HD: Hemodialysis
HIC: High income country
ISN: International Society of Nephrology
KF: Kidney failure
KRT: Kidney replacement therapy
LIC: Low income country
LMIC: Lower middle income country
NIS: Newly Independent States
PAD: Peripheral arterial disease
OSEA: South East Asia
UMIC: Upper middle income country
