# Supplementary material for: Global variations in funding and use of hemodialysis accesses: an international report using the ISN Global Kidney Health Atlas
Source: BMC Nephrol. 2024 May 8;25:159. doi: 10.1186/s12882-024-03593-z (PMC11080121; doi:10.1186/s12882-024-03593-z)
Supplement: Supplementary file 1 — Supplementary Material 1 [file 12882_2024_3593_MOESM1_ESM.docx]

**Additional Figure 1**

**
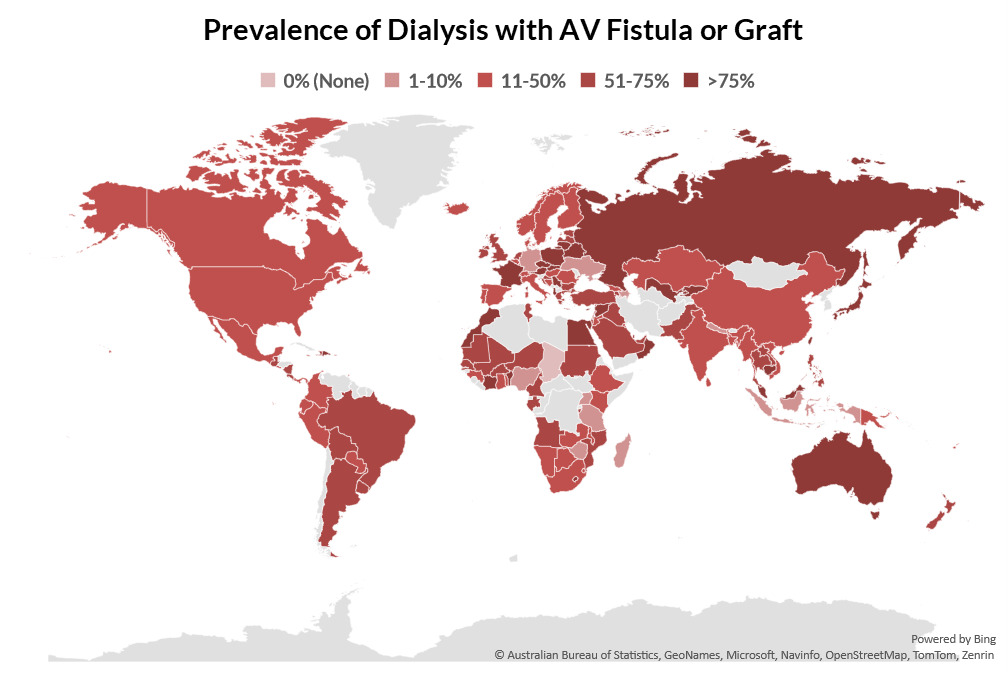
**

Supplemental Figure 1. Geographic heatmap for responses to survey question “For hemodialysis, what proportion of prevalent patients dialyze with a functioning vascular access (AV fistula or graft)?” Answers range from 0% (None), 1-10% (Few), 11-50% (Some), 51-75% (Most), and >75% (Almost all). AV, arteriovenous.

**Additional Figure 2**

**
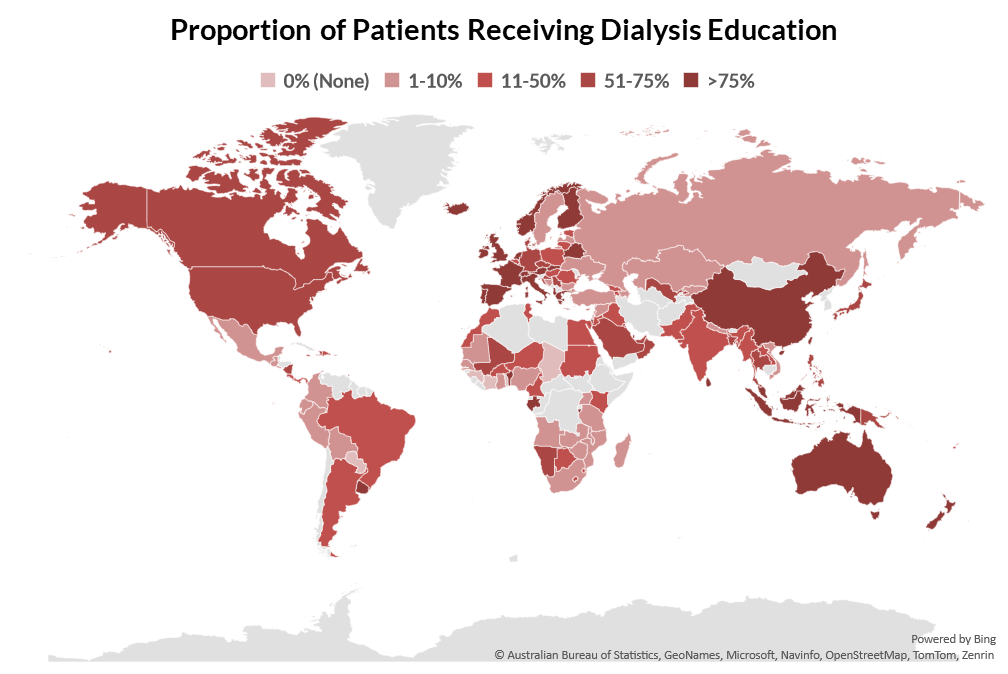
**

Supplemental Figure 2. Geographic heatmap for responses to survey question “For either hemodialysis or peritoneal dialysis, what proportion of patients routinely receive education about the best means of access and timely surgery (for example, six months before start of hemodialysis, one month before start of peritoneal dialysis)?” Answers range from 0% (None), 1-10% (Few), 11-50% (Some), 51-75% (Most), and >75% (Almost all).
