# Supplementary material for: Global variations in funding and use of hemodialysis accesses: an international report using the ISN Global Kidney Health Atlas
Source: BMC Nephrol. 2024 May 8;25:159. doi: 10.1186/s12882-024-03593-z (PMC11080121; doi:10.1186/s12882-024-03593-z)
Supplement: Supplementary file 2 — Supplementary Material 2 [file 12882_2024_3593_MOESM2_ESM.docx]

**Additional Table 1: Proportion of patients routinely starting dialysis with a functioning vascular access (AV fistula or graft): - (N, %)**

|  | 0% (None) | | 1-10% (Few) | | 11-50% (Some) | | 51-75% (Most) | | >75% (Almost all) | | unknown | | Total |
| --- | --- | --- | --- | --- | --- | --- | --- | --- | --- | --- | --- | --- | --- |
| Overall | 7 | (4) | 64 | (40) | 52 | (33) | 28 | (18) | 7 | (4) | 2 | (1) | 160 |
|  |  |  |  |  |  |  |  |  |  |  |  |  |  |
| ISN region: |  |  |  |  |  |  |  |  |  |  |  |  |  |
| Africa | 5 | (13) | 24 | (60) | 8 | (20) | 3 | (8) | 0 | (0) | 0 | (0) | 40 |
| Eastern & Central Europe | 0 | (0) | 3 | (19) | 9 | (56) | 4 | (25) | 0 | (0) | 0 | (0) | 16 |
| Latin America | 2 | (10) | 11 | (52) | 6 | (29) | 2 | (10) | 0 | (0) | 0 | (0) | 21 |
| Middle East | 0 | (0) | 4 | (36) | 5 | (45) | 1 | (9) | 1 | (9) | 0 | (0) | 11 |
| NIS & Russia | 0 | (0) | 2 | (20) | 4 | (40) | 2 | (20) | 1 | (10) | 1 | (10) | 10 |
| North America & the Caribbean | 0 | (0) | 9 | (75) | 2 | (17) | 1 | (8) | 0 | (0) | 0 | (0) | 12 |
| North & East Asia | 0 | (0) | 1 | (17) | 1 | (17) | 1 | (17) | 3 | (50) | 0 | (0) | 6 |
| OSEA | 0 | (0) | 9 | (56) | 5 | (31) | 1 | (6) | 0 | (0) | 1 | (6) | 16 |
| South Asia | 0 | (0) | 1 | (17) | 4 | (67) | 1 | (17) | 0 | (0) | 0 | (0) | 6 |
| Western Europe | 0 | (0) | 0 | (0) | 8 | (36) | 12 | (55) | 2 | (9) | 0 | (0) | 22 |
|  |  |  |  |  |  |  |  |  |  |  |  |  |  |
| World Bank Groups: |  |  |  |  |  |  |  |  |  |  |  |  |  |
| Low income | 3 | (17) | 9 | (50) | 4 | (22) | 1 | (6) | 1 | (6) | 0 | (0) | 18 |
| Lower-middle income | 3 | (7) | 24 | (57) | 9 | (21) | 3 | (7) | 1 | (2) | 2 | (5) | 42 |
| Upper-middle income | 1 | (3) | 19 | (51) | 12 | (32) | 4 | (11) | 1 | (3) | 0 | (0) | 37 |
| High income | 0 | (0) | 12 | (19) | 27 | (43) | 20 | (32) | 4 | (6) | 0 | (0) | 63 |

Abbreviations: AV, arteriovenous; ISN, International Society of Nephrology; NIS, Newly Independent States; N, number; OSEA, Oceania and South East Asia

###

**Additional Table 2: Proportion of patients routinely starting dialysis with a tunneled dialysis catheter: - (N, %)**

|  | 0% (None) | | 1-10% (Few) | | 11-50% (Some) | | 51-75% (Most) | | >75% (Almost all) | | unknown | | Total |
| --- | --- | --- | --- | --- | --- | --- | --- | --- | --- | --- | --- | --- | --- |
| Overall | 7 | (4) | 66 | (41) | 59 | (37) | 15 | (9) | 9 | (6) | 4 | (3) | 160 |
|  |  |  |  |  |  |  |  |  |  |  |  |  |  |
| ISN region: |  |  |  |  |  |  |  |  |  |  |  |  |  |
| Africa | 6 | (15) | 20 | (50) | 12 | (30) | 1 | (3) | 1 | (3) | 0 | (0) | 40 |
| Eastern & Central Europe | 0 | (0) | 8 | (50) | 7 | (44) | 1 | (6) | 0 | (0) | 0 | (0) | 16 |
| Latin America | 0 | (0) | 13 | (62) | 5 | (24) | 3 | (14) | 0 | (0) | 0 | (0) | 21 |
| Middle East | 0 | (0) | 5 | (45) | 4 | (36) | 1 | (9) | 1 | (9) | 0 | (0) | 11 |
| NIS & Russia | 1 | (10) | 5 | (50) | 3 | (30) | 0 | (0) | 0 | (0) | 1 | (10) | 10 |
| North America & the Caribbean | 0 | (0) | 1 | (8) | 4 | (33) | 1 | (8) | 6 | (50) | 0 | (0) | 12 |
| North & East Asia | 0 | (0) | 3 | (50) | 2 | (33) | 1 | (17) | 0 | (0) | 0 | (0) | 6 |
| OSEA | 0 | (0) | 6 | (38) | 6 | (38) | 1 | (6) | 1 | (6) | 2 | (13) | 16 |
| South Asia | 0 | (0) | 4 | (67) | 2 | (33) | 0 | (0) | 0 | (0) | 0 | (0) | 6 |
| Western Europe | 0 | (0) | 1 | (5) | 14 | (64) | 6 | (27) | 0 | (0) | 1 | (5) | 22 |
|  |  |  |  |  |  |  |  |  |  |  |  |  |  |
| World Bank Groups: |  |  |  |  |  |  |  |  |  |  |  |  |  |
| Low income | 3 | (17) | 8 | (44) | 7 | (39) | 0 | (0) | 0 | (0) | 0 | (0) | 18 |
| Lower-middle income | 2 | (5) | 24 | (57) | 10 | (24) | 3 | (7) | 1 | (2) | 2 | (5) | 42 |
| Upper-middle income | 2 | (5) | 19 | (51) | 10 | (27) | 2 | (5) | 4 | (11) | 0 | (0) | 37 |
| High income | 0 | (0) | 15 | (24) | 32 | (51) | 10 | (16) | 4 | (6) | 2 | (3) | 63 |

Abbreviations: ISN, International Society of Nephrology; NIS, Newly Independent States; N, number; OSEA, Oceania and South East Asia

**Additional Table 3: Proportion of patients commonly starting dialysis with a temporary dialysis catheter: - (N, %)**

|  | 0% (None) | | 1-10% (Few) | | | 11-50% (Some) | | | 51-75% (Most) | | | >75% (Almost all) | | | unknown | | | Total | |  |
| --- | --- | --- | --- | --- | --- | --- | --- | --- | --- | --- | --- | --- | --- | --- | --- | --- | --- | --- | --- | --- |
| Overall | 3 | (2) | | 41 | (26) | | 42 | (26) | | 31 | (19) | | 40 | (25) | | 3 | (2) | | 160 | |
|  |  |  | |  |  | |  |  | |  |  | |  |  | |  |  | |  | |
| ISN region: |  |  | |  |  | |  |  | |  |  | |  |  | |  |  | |  | |
| Africa | 1 | (3) | | 2 | (5) | | 6 | (15) | | 10 | (25) | | 20 | (50) | | 1 | (3) | | 40 | |
| Eastern & Central Europe | 0 | (0) | | 0 | (0) | | 10 | (63) | | 4 | (25) | | 2 | (13) | | 0 | (0) | | 16 | |
| Latin America | 0 | (0) | | 4 | (19) | | 3 | (14) | | 4 | (19) | | 10 | (48) | | 0 | (0) | | 21 | |
| Middle East | 0 | (0) | | 1 | (9) | | 6 | (55) | | 1 | (9) | | 3 | (27) | | 0 | (0) | | 11 | |
| NIS & Russia | 0 | (0) | | 3 | (30) | | 3 | (30) | | 2 | (20) | | 1 | (10) | | 1 | (10) | | 10 | |
| North America & the Caribbean | 2 | (17) | | 3 | (25) | | 3 | (25) | | 4 | (33) | | 0 | (0) | | 0 | (0) | | 12 | |
| North & East Asia | 0 | (0) | | 4 | (67) | | 0 | (0) | | 1 | (17) | | 1 | (17) | | 0 | (0) | | 6 | |
| OSEA | 0 | (0) | | 7 | (44) | | 4 | (25) | | 2 | (13) | | 3 | (19) | | 0 | (0) | | 16 | |
| South Asia | 0 | (0) | | 1 | (17) | | 3 | (50) | | 2 | (33) | | 0 | (0) | | 0 | (0) | | 6 | |
| Western Europe | 0 | (0) | | 16 | (73) | | 4 | (18) | | 1 | (5) | | 0 | (0) | | 1 | (5) | | 22 | |
|  |  |  | |  |  | |  |  | |  |  | |  |  | |  |  | |  | |
| World Bank Groups: |  |  | |  |  | |  |  | |  |  | |  |  | |  |  | |  | |
| Low income | 1 | (6) | | 1 | (6) | | 3 | (17) | | 3 | (17) | | 9 | (50) | | 1 | (6) | | 18 | |
| Lower-middle income | 0 | (0) | | 5 | (12) | | 11 | (26) | | 9 | (21) | | 16 | (38) | | 1 | (2) | | 42 | |
| Upper-middle income | 1 | (3) | | 6 | (16) | | 8 | (22) | | 11 | (30) | | 11 | (30) | | 0 | (0) | | 37 | |
| High income | 1 | (2) | | 29 | (46) | | 20 | (32) | | 8 | (13) | | 4 | (6) | | 1 | (2) | | 63 | |

Abbreviations: ISN, International Society of Nephrology; NIS, Newly Independent States; N, number; OSEA, Oceania and South East Asia

**Additional Table 4: Dominant dialysis initiation approach - (N, %)**

|  | AV Fistula or Graft (AVF/AVG) | | Tunneled Dialysis Catheter | | Temporary Dialysis Catheter | | Co-Dominant AVF/AVG and Tunneled | | Co-Dominant Tunneled and Temporary | | Co-Dominant AVF/AVG and Temporary | | Relatively Equal Use | | Total |
| --- | --- | --- | --- | --- | --- | --- | --- | --- | --- | --- | --- | --- | --- | --- | --- |
| Overall | 35 | (23) | 21 | (14) | 67 | (43) | 5 | (3) | 6 | (4) | 11 | (7) | 10 | (6) | 155 |
|  |  |  |  |  |  |  |  |  |  |  |  |  |  |  |  |
| ISN region: |  |  |  |  |  |  |  |  |  |  |  |  |  |  |  |
| Africa | 2 | (5) | 2 | (5) | 29 | (76) | 0 | (0) | 2 | (5) | 1 | (3) | 2 | (5) | 38 |
| Eastern & Central Europe | 4 | (25) | 1 | (6) | 6 | (38) | 0 | (0) | 0 | (0) | 2 | (13) | 3 | (19) | 16 |
| Latin America | 3 | (14) | 3 | (14) | 14 | (67) | 1 | (5) | 0 | (0) | 0 | (0) | 0 | (0) | 21 |
| Middle East | 2 | (18) | 1 | (9) | 4 | (36) | 0 | (0) | 1 | (9) | 2 | (18) | 1 | (9) | 11 |
| NIS & Russia | 3 | (33) | 0 | (0) | 3 | (33) | 0 | (0) | 0 | (0) | 2 | (22) | 1 | (11) | 9 |
| North America & the Caribbean | 1 | (8) | 7 | (58) | 4 | (33) | 0 | (0) | 0 | (0) | 0 | (0) | 0 | (0) | 12 |
| North & East Asia | 4 | (67) | 0 | (0) | 1 | (17) | 0 | (0) | 1 | (17) | 0 | (0) | 0 | (0) | 6 |
| OSEA | 3 | (20) | 3 | (20) | 4 | (27) | 1 | (7) | 1 | (7) | 0 | (0) | 3 | (20) | 15 |
| South Asia | 1 | (17) | 0 | (0) | 2 | (33) | 0 | (0) | 0 | (0) | 3 | (50) | 0 | (0) | 6 |
| Western Europe | 12 | (57) | 4 | (19) | 0 | (0) | 3 | (14) | 1 | (5) | 1 | (5) | 0 | (0) | 21 |
|  |  |  |  |  |  |  |  |  |  |  |  |  |  |  |  |
| World Bank Groups: |  |  |  |  |  |  |  |  |  |  |  |  |  |  |  |
| Low income | 1 | (6) | 1 | (6) | 12 | (75) | 0 | (0) | 1 | (6) | 0 | (0) | 1 | (6) | 16 |
| Lower-middle income | 4 | (10) | 5 | (13) | 24 | (60) | 0 | (0) | 0 | (0) | 5 | (13) | 2 | (5) | 40 |
| Upper-middle income | 6 | (16) | 4 | (11) | 20 | (54) | 0 | (0) | 2 | (5) | 3 | (8) | 2 | (5) | 37 |
| High income | 24 | (39) | 11 | (18) | 11 | (18) | 5 | (8) | 3 | (5) | 3 | (5) | 5 | (8) | 62 |

Abbreviations: AV, arteriovenous; ISN, International Society of Nephrology; NIS, Newly Independent States; N, number; OSEA, Oceania and South East Asia

**Additional Table 5: Survey participants**

|  | **Low-income** | **Lower-middle-income** | **Upper-middle-income** | **High-income** |
| --- | --- | --- | --- | --- |
| AFRICA | Burkina Faso | Angola | Botswana |  |
|  | Burundi | Benin | Gabon |  |
|  | Central African Republic | Cameroon | Mauritius |  |
|  | Chad | Cape Verde | Namibia |  |
|  | Congo, Dem. Rep. | Congo, Rep. | South Africa |  |
|  | Ethiopia | Cote d'Ivoire |  |  |
|  | Gambia | Egypt |  |  |
|  | Guinea | Ghana |  |  |
|  | Madagascar | Kenya |  |  |
|  | Malawi | Lesotho |  |  |
|  | Mali | Mauritania |  |  |
|  | Mozambique | Morocco |  |  |
|  | Niger | Nigeria |  |  |
|  | Somalia | Senegal |  |  |
|  | Sudan | Swaziland |  |  |
|  | Togo | Tanzania |  |  |
|  | Uganda | Tunisia |  |  |
|  | Zambia | Zimbabwe |  |  |
|  |  |  |  |  |
| EASTERN & CENTRAL EUROPE |  |  | Albania | Croatia |
|  |  |  | Bosnia and Herzegovina | Cyprus |
|  |  |  | Bulgaria | Czech Republic |
|  |  |  | Macedonia, FYR | Estonia |
|  |  |  | Serbia | Hungary |
|  |  |  | Turkey | Latvia |
|  |  |  |  | Lithuania |
|  |  |  |  | Poland |
|  |  |  |  | Romania |
|  |  |  |  | Slovak Republic |
|  |  |  |  |  |
| LATIN AMERICA |  | Bolivia | Argentina | British Virgin Islands |
|  |  | El Salvador | Brazil | Cayman Islands |
|  |  | Haiti | Colombia | Chile |
|  |  | Nicaragua | Costa Rica | Curaçao |
|  |  |  | Dominican Republic | Panama |
|  |  |  | Ecuador | Puerto Rico |
|  |  |  | Guatemala | Uruguay |
|  |  |  | Mexico |  |
|  |  |  | Paraguay |  |
|  |  |  | Peru |  |
|  |  |  | Venezuela |  |
|  |  |  |  |  |
| THE MIDDLE EAST | Syrian Arab Republic | Iran, Islamic Rep. | Iraq | Kuwait |
|  |  | Lebanon | Jordan | Oman |
|  |  | West Bank and Gaza |  | Qatar |
|  |  |  |  | Saudi Arabia |
|  |  |  |  | United Arab Emirates |
|  |  |  |  |  |
| NEWLY INDEPENDENT STATES & RUSSIA |  | Kyrgyz Republic | Armenia |  |
|  |  | Tajikistan | Azerbaijan |  |
|  |  | Ukraine | Belarus |  |
|  |  | Uzbekistan | Georgia |  |
|  |  |  | Kazakhstan |  |
|  |  |  | Russian Federation |  |
|  |  |  |  |  |
| NORTH & EAST ASIA |  |  | China | Hong Kong |
|  |  |  |  | Japan |
|  |  |  |  | Korea, Rep. |
|  |  |  |  | Macao SAR, China |
|  |  |  |  | Taiwan |
|  |  |  |  |  |
| NORTH AMERICA & THE CARIBBEAN |  |  | Jamaica | Antigua and Barbuda |
|  |  |  | St. Lucia | Aruba |
|  |  |  | St. Vincent and the Grenadines | Bahamas |
|  |  |  |  | Barbados |
|  |  |  |  | Bermuda |
|  |  |  |  | Canada |
|  |  |  |  | Trinidad and Tobago |
|  |  |  |  | Turks and Caicos Islands |
|  |  |  |  | United States |
|  |  |  |  |  |
| OCEANIA & SOUTH EAST ASIA |  | Cambodia | American Samoa | Australia |
|  |  | Indonesia | Fiji | Brunei Darussalam |
|  |  | Lao PDR | Malaysia | New Caledonia |
|  |  | Myanmar | Thailand | New Zealand |
|  |  | Papua New Guinea |  | Singapore |
|  |  | Philippines |  |  |
|  |  | Samoa |  |  |
|  |  | Solomon Islands |  |  |
|  |  | Vanuatu |  |  |
|  |  | Vietnam |  |  |
|  |  |  |  |  |
| SOUTH ASIA | Afghanistan | Bangladesh | Maldives |  |
|  |  | Bhutan |  |  |
|  |  | India |  |  |
|  |  | Nepal |  |  |
|  |  | Pakistan |  |  |
|  |  | Sri Lanka |  |  |
|  |  |  |  |  |
| WESTERN EUROPE |  |  |  | Andorra |
|  |  |  |  | Austria |
|  |  |  |  | Belgium |
|  |  |  |  | Denmark |
|  |  |  |  | Finland |
|  |  |  |  | France |
|  |  |  |  | Germany |
|  |  |  |  | Greece |
|  |  |  |  | Iceland |
|  |  |  |  | Ireland |
|  |  |  |  | Israel |
|  |  |  |  | Italy |
|  |  |  |  | Liechtenstein |
|  |  |  |  | Luxembourg |
|  |  |  |  | Malta |
|  |  |  |  | Netherlands |
|  |  |  |  | Norway |
|  |  |  |  | Portugal |
|  |  |  |  | Spain |
|  |  |  |  | Sweden |
|  |  |  |  | Switzerland |
|  |  |  |  | United Kingdom |
